# A Genetic Neural Net Model for the Relationship between Pre-School and Attention in Early Childhood

**DOI:** 10.1155/2022/6451199

**Published:** 2022-06-06

**Authors:** Liping Wang, Na Yao

**Affiliations:** Tangshan Normal University, Tangshan 063000, Hebei, China

## Abstract

This paper uses an algorithm of a genetic neural network to conduct an in-depth study and analysis of the attention relationship in pre-school education of young children. In face expression images, expression features are often concentrated and distributed in some local regions, while convolutional neural networks are homogeneous for feature extraction of facial expression images. To address the problem of the nonuniform distribution of expression features, a facial expression recognition method based on the spatial domain attention mechanism is proposed. A comparison of the differences between group and grade before the intervention using a two-way multivariate ANOVA revealed a nonsignificant interaction effect with a value of 0.90 for the group-grade interaction effect and an *F*-value of 0.36. The group's main effect had a value of 0.90 and an *F*-value of 0.36, with the experimental and control groups being equal groups on multiple dependent variables. The salient features in face expression images that are more important for facial expression recognition can be selected adaptively, and relatively high weights are assigned to these salient features. Experimental results on the image library show that the introduction of a spatial domain attention mechanism can improve the average recognition accuracy of face expressions by about 1.1% and 1.0%, respectively. A hybrid domain attention mechanism-based face expression recognition method is proposed to extract important expression features from both the spatial domain and the channel domain. Experimental results on the image library show that combining the spatial domain attention mechanism and the channel domain attention mechanism can improve the average recognition accuracy by about 2.1% and 1.5%, respectively. The use of rule games was effective in developing children's sustained and selective attention; the effect of rule games on the sustained and selective attention of older boys and girls did not show significant gender differences.

## 1. Introduction

At every moment in our daily lives, there is an influx of information to our senses, and the accurate use of attention to process important information is an important part of young children's development and learning. The whole process of the experiment was carried out without the knowledge of the children. Attention is the primary condition for young children to perceive the world and access information, and they need to make connections with external information [1]. Many skills are shaped by the ongoing development of attention, and a young child with attention problems will have limited development in many areas, such as language development, problem-solving, and complex play [2]. Some studies have found that young children with severe attention deficits also have significantly lower intellectual development than their peers, and conversely, children with more focused attention also have better cognitive and social development. Attention is also a key factor in a person's success in doing and learning, and it has a significant impact on development throughout life, starting in the early years of school [3]. Attention plays an important role in the development of young children, so as a pre-school graduate student, it is important to study the attention of young children from a pre-school perspective. However, many parents and teachers find that when young children first enter primary school, they are prone to problems of inattention, drifting off in class, and dawdling in their homework, and they struggle with this. This is due to the inconsistency between the attention span required in primary school and kindergarten, as an activity in older classes lasts 25–30 minutes, while a class in primary school lasts 40 minutes. This is far from the attention span required in the classroom [4]. For older children to be better prepared for primary school and beyond, attention development should emphasize in the older years.

The early years are a critical period in children's attention development, and attention development is essential for their future growth. Attention development helps children to perceive the external world and to acquire information about it. It is a key factor in the development of learning habits and learning efficiency; it affects children's future learning and memory and studies have shown that attention deficits correlate with poorer academic performance [5]. At the same time, the level of attention development of young children affects their adaptation to the environment and social interactions, and the development of their attention level plays a very important role in their social development.

The advent of the big data era, accompanied by the problem of information overload, has made recommendation systems increasingly popular. Finally, 13 kinds of rule games were selected or adapted to generate the following rule game activity plan. The traditional matrix decomposition method is the most widely used model in collaborative filtering algorithms due to its characteristics such as high scalability of the algorithm and better predictive capability. However, in the big data environment, many contextual factors have become extremely easy to obtain, such as user demographics, weather, time, location, social networks, and so on. As a variety of contextual features are introduced into the model, extensions to traditional matrix decomposition methods are rapidly being developed to enable the introduction of more contextual factors into the model. However, most decomposition methods based on contextual features have their specific application scenarios, which greatly reduce the generalization capability of the algorithm compared to traditional matrix decomposition models. By comparing and analyzing the data of three different story videos at each repetition, the relationship between the number of story repetitions, the timing of the stories, the children's attentional characteristics, and the changes in their attention during the repetition of the story videos are explored. This study will provide a reference strategy for early childhood educators when implementing story video repetition. Through experimentation and observation, we can obtain the appropriate intervals of story repetition for older children and the effects of story timing and children's attentional characteristics on children's repetition of story videos and make recommendations to help teachers organize story teaching activities more scientifically and rationally.

## 2. Related Works

Feature extraction methods can be divided into two approaches: manual feature extraction and deep feature extraction. Manual feature extraction is based on the researcher's knowledge and understanding of facial expressions to describe an important area in the face image and extracts the expression features contained in the face image through a series of simple operations designed artificially; deep feature extraction is mainly used to invisibly extract the more abstract expression features contained in the face image using convolutional neural networks. The principal component analysis algorithm is one of the most used methods to globally extract features, retaining as few important features as possible from the original features and reducing the correlation between different classes of features. The difficulty factor of the task at this stage is relatively high. However, when using the PCA method, the two-dimensional image matrix must first be transformed into a one-dimensional vector, which can reduce the efficiency of face expression classification. To address the problems of the PCA algorithm for processing image data, Wakschlag et al. proposed an expression feature extraction method based on independent component analysis, which can extract the implicit feature information in face expression images more effectively [6]. Martzog et al. proposed a face expression feature extraction method based on multifeature fusion weighted principal component analysis [7]. Meyer et al. proposed a general expression sparse representation and classification lexicon building method, where the subject's facial expression images were subtracted from the neutral face images of the same subject when training the model to simulate the variation of each type of facial expression, and the test images sparsely represented as linear combinations of the six basic facial expression principal components when performing face expression classification [8].

The breadth of attention is the number of objects that children can grasp at the same instant, typically in the range of 2–3 for young children and 4–6 for adults. During the activity, children's attention span becomes relatively larger if the target objects are arranged in a regular pattern and smaller for irregular arrangements or cluttered presentations. If the target objects are the same color, children's attention span is generally larger than if they are complex; if the target objects are the same size, children's attention span is larger than if they are not the same size. Attention allocation refers to children's ability to focus their attention on two or more objects at the same time, and children are much less able than adults to allocate their attention [9]. Adults can talk and laugh while eating a snack or even continue to do what they are doing in their hands, whereas in the same situation, young children generally focus only on eating the snack, or if they need to express themselves, they stop the activity they are doing or put down the object they are holding and focus on talking or listening [10]. One of the main characteristics of early childhood stage activities is their love of movement. Because of their neurological characteristics of excitement over inhibition, young children have poor control over their attention and are easily distracted to the extent that they cannot focus their attention on the object they should be paying attention to for long periods [11]. However, if the activity is of interest to the child, and if it is done appropriately, the child's attention will remain somewhat stable.

It takes longer for children to complete the task, and there are more interference stimuli, so the game activities that choose attention are also more difficult. For these reasons, the development of a good attention span in the older years is both practical and scientific. Some scholars have suggested that rule games can effectively promote children's attention development. Therefore, the author attempts to investigate the effect of rule games on the development of children's attention in the older classes by using rule games as the content of the activities and adopting an empirical research paradigm to investigate the effect of rule games on children's attention in the older classes. This study will examine the effect of rules play on the attention of older children in conjunction with an empirical research paradigm. The study will not only provide data to support the development of attention in older children but also provide examples and ideas for kindergartens to implement similar games scientifically and raise more attention to the development and cultivation of attention in pre-schools by teachers and parents.

## 3. The Design of an Attentional Genetic Neural Network Model

The idea of attentional mechanisms, first proposed by experts in the field of imagery, is based on the idea that humans, when perceiving the outside world, selectively focus on only some of the information features of interest rather than distributing attention evenly across the scene. The powerful learning capability of neural networks often relies on many training samples, and complex network structures are often accompanied by large parameter systems, a process that often results in information overload problems. To alleviate such problems, the attention mechanism can be used to focus learning on a small but critical number of components when dealing with large amounts of data information. The GCN is mainly used to learn the hierarchical information of the samples, while the word order information and context in the text are also extremely important for the relationship extraction task; for the problem of long-distance text dependency in RNN, the LSTM is introduced to learn text contextual features and other high-level semantic features [12]. The CA module proposed in this paper can more reasonably capture the important channels in the feature map tensor that play a key role in the facial expression recognition task. In this paper, we combine bidirectional long- and short-term memory networks and graph convolutional networks to model the relation extraction problem. While using BiLSTM to learn word order and long-range context-dependency information, we extract richer hierarchical information in the syntactic dependency tree through a shared filter of graph convolution.

**Figure 1:**
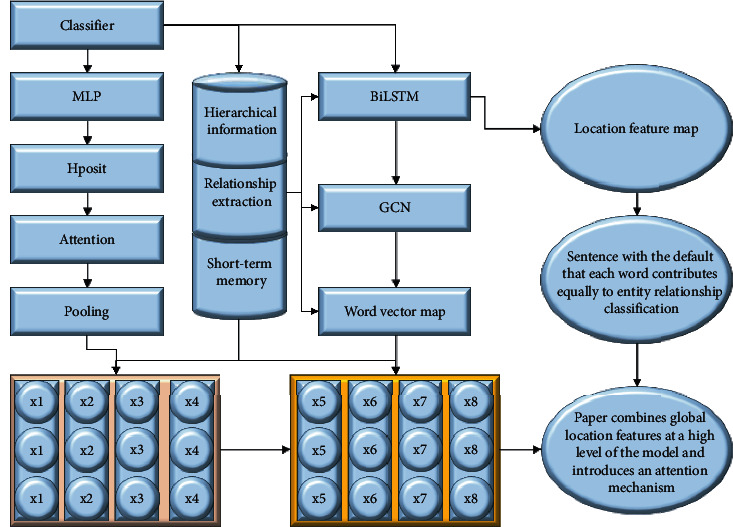
AL-GCN network framework diagram.

Unlike previous approaches that use word position information as word vector features at the input layer, this paper uses the word position information to model global semantic features. In addition, existing methods learn each word in a sentence with the default that each word contributes equally to entity relationship classification, but the words in the sentence have different influences on the classification results. In summary, this paper combines global location features at a high level of the model and introduces an attention mechanism to learn the different contributions of different words to the predicted relationships between entities separately and proposes a method to extract the relationships between entities by integrating BiLSTM, GCN, and location attention mechanism, called AL-GCN model, as shown in Figure 1.

The LBP algorithm is a binary to decimal conversion; the decimal LBP value is often limited to a power of 2. If the power of 2 assignment changed to a network weight assignment, it can be extended to a generalized LBP from a network perspective. Therefore, borrowing the idea of traditional LBP, the binary convolution filter with Bernoulli distribution is used to convolve the whole image, and then the results of each filter are summed up, and the calculation is as follows:

(1) $$\begin{array}{c} \sum_{i=1}^{n}\partial \left(b_{i}\times x\right)v_{i}^{2}, \end{array}$$

(2) $$\begin{array}{c} L_{p}\left(x_{i},y_{j}\right)={\left(\sum_{l=1}^{n}{\left|x_{i}^{l}+x_{j}^{l}\right|}^{p}\right)}^{p}. \end{array}$$

The pixel gradient value reflects the trend of the image texture information (edge); the gradient of the image *I* at point (*x*, *y*) contains the size and direction vector; and the gradient expression is shown in the following equation:

(3) $$\begin{array}{c} G=\sqrt{G_{x}^{2}-G_{y}^{2}}. \end{array}$$

In this section, the gradient information is used to obtain the texture change trend, and the LBP dynamic sampling radius strategy is designed to divide the small gradient sampling radius (R_Small G) and large gradient sampling radius (R_Big G) regions according to the gradient value difference. The delineation was based on the GMM model with a probability distribution model.

(4) $$\begin{array}{c} P\left(x\right)=\sum_{k=1}^{K}w_{k}g\left(x^{2}|\mu_{k}^{2}-\sum k^{2}\right). \end{array}$$

The spatial attention (SA) mechanism is a soft attention mechanism approach, which is designed to use a mask formation mechanism to compute a weight mask that identifies important features and allows the convolutional neural network to efficiently learn the region of interest, thus mapping the spatial information in the original image to another space to retain the important features in the image. The spatial domain attention mechanism is represented by the spatial transformation network (STN), which improves the performance of a convolutional neural network by spatially transforming the feature map tensor to extract key information and reduce the impact of minor features in the feature map tensor on the convolutional neural network [13]. Since the useful features contained in each channel in the feature map tensor are not the same, the use of the CA module can effectively weaken or suppress the adverse effects and interference of the features on the redundant channels in the feature map tensor on facial expression recognition. The STN can be used as a stand-alone module in a convolutional neural network, which can insert an arbitrary number of nodes at different nodes in different convolutional neural networks, thus enabling the spatial transformation of the feature map tensor at different depths without increasing the computational effort of the convolutional neural network.

Because the attention mechanism has fewer parameters, the serial structure is not much different from the parallel structure in terms of computational time consumption, and the serial structure has more nonlinear activation functions superimposed to obtain better nonlinear learning ability, so this paper designs a serial hybrid domain attention mechanism HA module by combining the SA module and CA module.

(5) $$\begin{array}{c} D_{s}=\left\{\left(u,i,j\right)|i\in I_{u}^{+}\wedge j\in I\backslash I_{u}^{-}\right\}. \end{array}$$

Pairwise learning is an important technique for personalized ranking with implicit feedback. Assuming that each user is more interested in previously selected items than others, pairwise learning methods can learn user preferences not only from observed user feedback but also from potential interactions between users and items. However, many training examples are randomly derived based on such assumptions, which make the learning process often slow to converge and even leads to poor predictive models [14]. In addition, the cold-start problem often plagues pairwise learning approaches, as most traditional methods in the personalized ranking only consider explicit scoring or implicit feedback.

**Figure 2:**
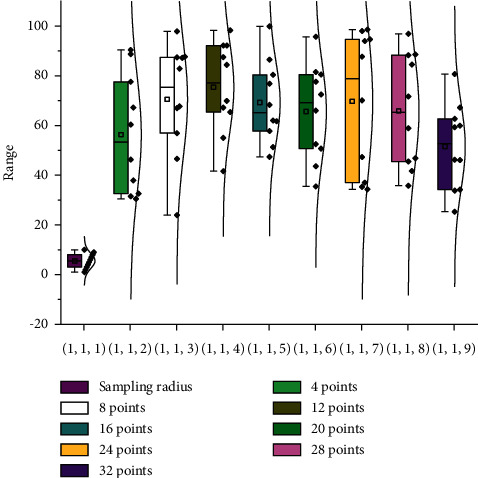
Comparison of the effect of different sampling points and sampling radius.

The LSTM used in the relationship extraction task will process the text in word-by-word order and obtain the hidden vector by transforming the neurons in the hidden layer. However, in the process of generating the hidden layer vector, on the one hand, the words near the end of the text will dominate, creating an information bias problem; on the other hand, each word only has an impact on the subsequent words, and each word feature can only capture the information above it when it learned. There are many activities that support the development of attention in pre-school to be feasible and effective, so there is theoretical support for the development of sustained attention through rule games. The samples used in the relational extraction task are strongly correlated, and a unidirectional LSTM is unable to meet the learning requirements of relational extraction. The bidirectional LSTM unit consists of two independent LSTM units, where the forward LSTM unit encodes the text sequence from front to back and the backward LSTM unit encodes the text sequence from back to front. The hidden vectors output from the same time step of the two LSTM units are stitched together to form the output vector, as shown in Figure 2.

Since the Oulu-CASIA expression library only has the effect of lighting changes on facial expressions and the LBP algorithm is more robust to lighting changes, using the Oulu-CASIA expression library as the basic library for LBP sampling point comparison experiments can avoid the effect of lighting on the experimental results. Region extraction makes the experiment more convincing [15]. Content validity refers to the appropriateness of the test items to sample the target content or behavior. This may be related to the pixel sampling coverage of the expression region, where a sampling radius of four pixels in the set with small pixel gradient values can better cover the smooth texture region and a sampling radius of two pixels in the set with large pixel gradient values for larger sets of pixel gradients. In summary, the LRS algorithm was set to eight sampling points in the experiments, and the sampling radius was dynamically selected in the range (2, 2, 4).

## 4. Model Design of the Relationship between Pre-School Education and Attention in Early Childhood

In this study, the scale was developed by first designing an open-ended interview outline, conducting expert interviews with frontline teachers and graduate students and teachers in related disciplines, collecting relevant attentional behavioral performance of young children, preparing the first draft with literature, conducting expert ratings, inviting frontline teachers to rate the degree of compliance of the items, and forming a formal administration questionnaire through item analysis [16].

In psychology, attention is defined as the ability of a person's mental activity to be directed and focused on something. The change in attention in this study refers specifically to the change from focused to distracted attention during the repeated viewing of the story video in the kindergarten classroom. Secondly, in terms of the experimenter, to avoid the influence of the researcher on the experiment, the researcher entered the subject's class as a new teacher at the beginning of the new term and only started the pre-experiment after a month of mutual familiarity with the subject [17].

**Figure 3:**
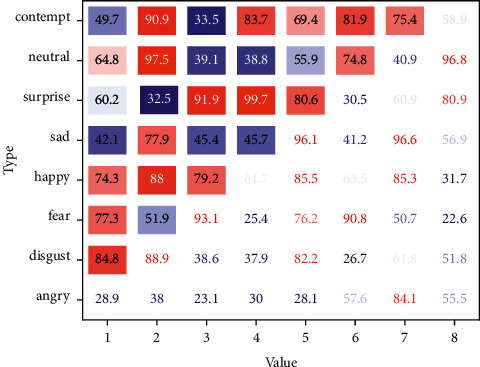
Confusion matrix for the recognition of the expression bank.

Finally, the experiment was conducted without the children's knowledge to avoid the novelty of the experiment itself. When compiling items of the scale, referring to the results of previous studies and expert interviews, the items are logically analyzed, and some items are modified or deleted based on the actual performance of children's attention. The camera was set up in front of the activity room to take pictures of the children during the kindergarten's national day activities, and the novelty of the camera was minimized by turning it on before each activity for a week before the camera entered the room. One week later, the video recording of the experiment began without the subjects' knowledge. Each experiment was conducted at the same time and in the same physical environment as the activity room, as shown in Figure 3.

The final instrument was then administered to a large group of teachers who were asked to assess the behavioral performance of the students and to analyze the quality of the items after the formal administration. Rule-based games promote sustained and selective attention in older female children more than in male children. This hypothesis is based on the finding that the level of attentional stability was significantly higher in girls than in boys before and after the maze training experiment and that the maze game is a type of rule game.

During the formal experiment, the researcher participated fully in the experiment, watching the story video with the subjects in a fixed position during each experiment, without interfering at all in the process of the experiment, to restore the most realistic situation of the subjects. At the same time, to avoid experimental errors caused by the interaction effect between different children, the position of the subjects was fixed for each experiment, as was their relative position to the researcher, and the number of participants in the experiment was also fixed (except in the case of individual subjects who were unable to participate in the experiment for special reasons). Furthermore, before the start of each experiment, the researcher organized a group toilet and water break and reminded the children to change their clothing and so on to avoid the physiological needs associated with the experiment from affecting the process.

The design of this rule game activity framework selected some classic rule games in kindergartens and some rule games mentioned in some papers and initially selected 25 rule games that meet the developmental characteristics and interest needs of 5–6-year-olds, then modified the original scheme based on the above design ideas, the basic principles followed, and the specific selection and adaptation criteria, as well as discussions with psychology experts and front-line kindergarten teachers. The intellectual development of children with severe attention deficit is also significantly lower than that of children of the same age. Pre-study visits to the kindergarten resulted in the selection or adaptation of 13 rule games (including educational, musical, and sports games), resulting in the following rule game activity program [18]. The series of rule games were played in a sequence from easy to difficult, and the 15 rule game activities in this study were based on events that occurred in the number kingdom to make the games more interesting. Each play activity in phases 1 and 2 consisted of one rule game, and in phase 3, because of the goal of further development, each play activity consisted of one previously played rule game plus a new rule game, which were connected by a story situation.

**Figure 4:**
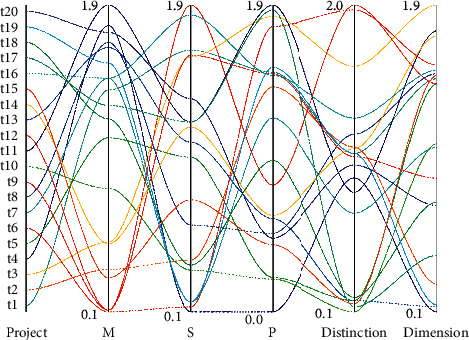
Item analysis of the formal scale.

According to the Clinical Model of Attention, we know that higher levels of attention are based on lower levels of attention, so the first stage is to develop sustained attention so that attention that requires more cognitive resources can develop better if the foundation of the lower levels of attention firmly established. The goal at this stage is therefore to develop sustained attention. Selective attention tasks begin to be added at this stage. This stage aims to promote the initial development of sustained and selective attention, so rule-based play activities are chosen to meet the goals of this stage [19]. Conversely, the cognitive and social development of children with more concentrated attention is also better. The difficulty factor for tasks in this stage is higher, as children need to last longer to complete the task, and there are more distracting stimuli making the selection of attentional play activities more difficult. After the first two stages of practice, children can adapt to the more difficult tasks, allowing them to further develop sustained attention, selective attention, and other attention (mainly to exercise their cognitive flexibility), as shown in Figure 4.

The item means for the scale ranged from 1.66 to 2.20, with no ceiling or floor effects; the standard deviations ranged from 0.73 to 0.87, which was more evenly distributed, so there was no specificity in the items. The total question correlations for the items ranged from 0.61 to 0.79, with mean discrimination of 0.74, all above 0.6 and significant at the 0.01 level. The correlations between the items and the dimension to which they belonged were all above 0.7 and significant at the 0.01 level.

This activity aims to develop persistent and selective attention. The rules of the game are simple, that is, find the target color bean among many different colors and put them together, thus fostering selective attention and sustained attention as the children focus on the game throughout. The children were very involved in the implementation of the game, and they were very attentive to the process of putting the pieces together. However, many parents and teachers have found that when children first enter primary school, they are prone to inattention, distraction in class and dawdling in homework.

## 5. Results and Analysis

### 5.1. Analysis of Algorithm Performance Results

The accuracy of the spatial domain attention-based convolutional neural network model for facial expression recognition on both the RAF-DB and FER2013 face expression image repositories improved over the improved convolutional neural network model, indicating that the maximum pooling and average pooling in the spatial domain attention SA module can effectively learn discriminative global and local features from facial expression images and accurately calculate the weights at each spatial location in the feature map tensor, thus reinforcing the role of important spatial features in the feature map tensor in the facial expression recognition task. Meanwhile, SA-ResNet-50 achieves the highest face expression recognition rate on both image libraries, which indicates that as the depth of the convolutional neural network increases, the use of the residual module can be more effective in extracting rich deep features, effectively alleviating the problem of gradient disappearance in the deep network model, and enhancing the generalization capability of the network.

**Figure 5:**
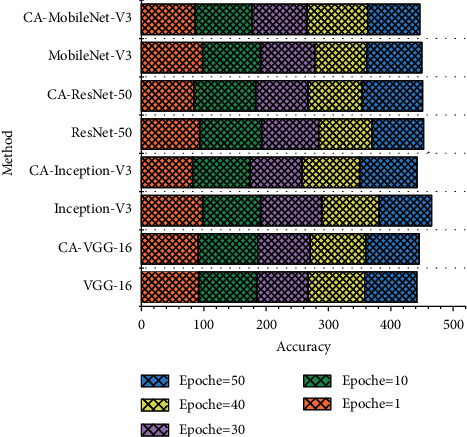
Experimental results of face expression image library.

In this paper, each convolutional neural network model is trained using the RAF-DB and FER2013 face expression image libraries, and the effect of the CA module of the channel domain attention mechanism on the recognition rate of facial expressions is investigated. In addition, the performance of the CA module was further validated by adding a comparison experiment between MobileNet-V3 and CA-MobileNet-V3, and the results are shown in Figure 5.

The convolutional neural network model based on the channel domain attention mechanism improved the accuracy of facial expression recognition on both the RAF-DB and FER2013 face expression image libraries compared to the improved convolutional neural network model. The experimental results show that the channel domain attention CA module can accurately calculate the weights of each channel in the feature map tensor, thus enhancing the role of important channel features in the feature map tensor in the facial expression recognition task and making the expression classification more accurate. Young children's connection with outside information must be done through attention. The level of children's attention development is related to whether the courses taught by kindergarten teachers can achieve the teaching purpose. Although the accuracy of MobileNet-V3 is lower than other network models, the model size of MobileNet-V3 is only 12 MB, which is much smaller than the 283 MB of ResNet-50, and the training time is less. It is also found that CA-MobileNet-V3 has a small performance improvement over MobileNet-V3 for both facial expression image libraries, which indicates that the proposed CA module can capture the important channels in the feature map tensor that are critical to the facial expression recognition task in a more reasonable way than the SE module. Since the feature tensor does not contain the same number of useful features on each channel, the use of the CA module can effectively weaken or suppress the negative impact and interference of features on redundant channels in the feature tensor on facial expression recognition and help improve the network model to focus on key expression features, thus helping to improve the accuracy of facial expression recognition.

**Figure 6:**
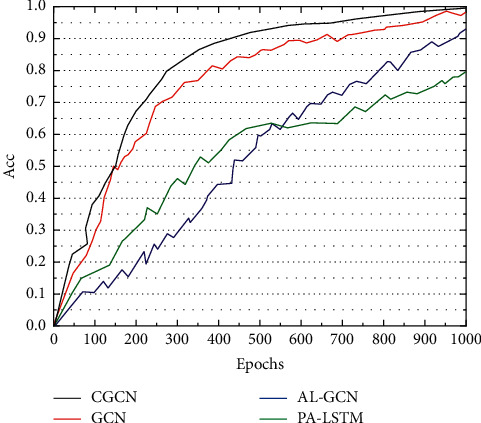
Trend of model F1 values on dev with increasing epoch.

The proposed AL-GCN model in this paper tends to converge to a higher F1 value than the other three models, demonstrating that AL-GCN can capture more information that is important for relationship extraction. Although PA-LSTM showed good performance on the training set upfront, the F1 value in the test set was only 65.1%, while the model AL-GCN in this paper achieved an F1 value of 67.6% on the test set, which was 2.5% higher than PA-LSTM, indicating that the generalization ability of the AL-GCN model was stronger than that of the PA-LSTM model, as shown in Figure 6.

The F1 values of most neural models are higher than those of traditional machine learning methods, indicating that deep learning models based on neural networks can learn deeper semantic features on relation extraction tasks by learning from a large amount of training data. Among the neural network models, LSTM is the model that uses only long- and short-term memory networks for relationship extraction. Compared with CGCN, a model that fuses LSTM and GCN, the CGCN method has improved 4.2%, 3.4%, and 3.7% in accuracy, recall, and F1 values, respectively. However, in the big data environment, it becomes extremely easy to obtain many contextual factors, such as the user's demographic information, weather, time, location, social network, and so on. It shows that the graphical convolutional network can be used for effective learning in the dependent syntactic tree, and the introduction of this partial hierarchical information can compensate for the lack of feature learning in the LSTM model, and the features captured by the two partial models can complement each other.

In addition, the model in this paper introduces an attention mechanism to compensate for the lack of entity location information in the extracted text sequences by the graph convolutional neural network. Instead of using the same weights for all words, the importance of words is modeled by the sequence of semantic information and the global position of words and entities, which in turn filters the information that is more important for the relationship extraction task and ignores irrelevant information. The AL-GCN model in Figure 6 achieves a 2.2%, 0.2%, and 1.2% improvement in accuracy, recall, and F1 values, respectively, by introducing an attention mechanism. Not only did they obtain the highest precision, recall, and F1 values among all deep learning relationship extraction models, but they also achieved the highest recall and F1 values among models with both traditional methods and deep learning, increasing the relationship classification recall and F1 values to 63.5% and 67.6%.

### 5.2. Model Validation Results

The face expression recognition system can capture face images from the camera or use locally saved face images directly. When the user chooses to enable the camera function, the system will automatically call the computer's camera to capture; the face detection module will perform face detection on each frame of the video captured by the camera; then the face part will be size normalized and input to the expression recognition module for recognition; and finally, the recognition result and the predicted probability of each type of expression will be displayed in the interface. The samples used in the relation extraction task have a strong contextual correlation, and the one-way LSTM obviously cannot meet the learning requirements of relation extraction. When the user chooses to use the face image selection function, he or she selects an image from the computer, and then the face detection module detects the face area in the input image, normalizes the size of the captured face, and inputs it into the expression recognition module.

There is also individualization of the number of times that children focus on each repeated story video as a proportion of the number of trials. Content validity refers to the extent to which the test questions sample the target content or behavior appropriately. In developing the items for the scale, the items were logically analyzed concerning relevant research from previous studies and expert interviews, and some items were modified or deleted based on the actual performance of young children's attention. Expert professors in mental health, postgraduate scholars in pre-school education, and experienced front-line educators were invited to rate the items' degree of conformity and to eliminate items that did not meet the requirements.

**Figure 7:**
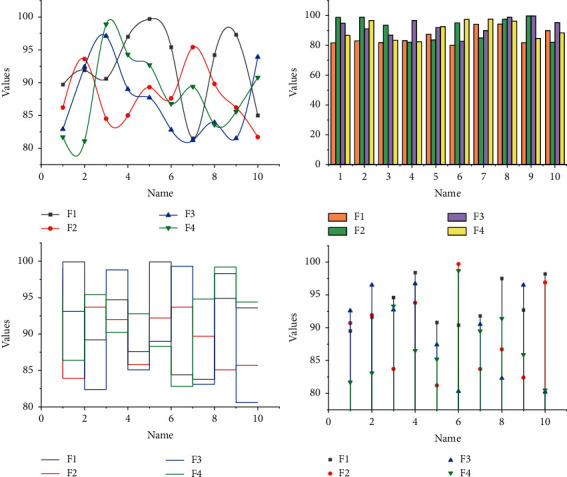
Correlation of teacher ratings.

The validity of the calibration correlations, as one of the indicators for measuring validity, mainly examines whether the predictions made by the test can be confirmed. In this study, teacher recommendations were selected as the school standard, and the results of the scale were correlated with teacher recommendations to examine the validity of the school standard. A total of 33 students in a kindergarten class were selected for this study and rated by their teacher for their attention and by their lead teacher for their daily performance in terms of whether they had attention problems. The two sets of data were correlated, and the results are shown in Figure 7. The correlation between the total scale and teacher recommendation was 0.57, and the correlation between the subscales and teacher recommendation ranged from 0.53 to 0.60 and were all significant at the 0.01 level.

In this study, an independent samples *T*-test was used to test whether the experimental and control groups were homogeneous before the experiment using a *p* < 0.05 protest. This was followed by a paired-sample *t*-test to analyze the effects of rule-based play on sustained and selective attention in the older children, using separate analyses of the differences between the pre- and post-test in the experimental group and the pre- and post-test in the control group. Finally, an independent samples *t*-test was used to determine whether there was a significant improvement in the experimental group of boys and girls.

**Figure 8:**
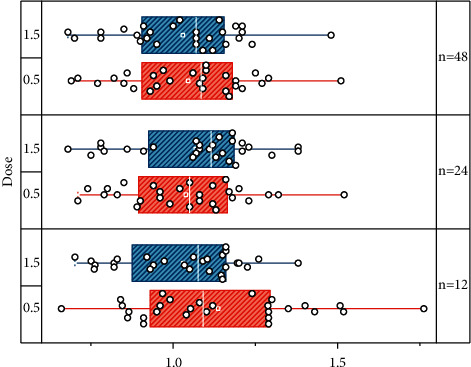
Comparative analysis of the mean scores of the experimental pre-test of attention of the children in the experimental and control groups.

To extract the word context information containing complete semantics, BiLSTM is used as the encoder here. An independent samples *t*-test was used to analyze whether there was a significant difference between the scores of the experimental group and the control group on the pre-test of sustained and selective attention. As can be seen from Figure 8, the scores for sustained attention (*p* = 0.417, *p* > 0.05) and selective attention (*p* = 0.698, *p* > 0.05), did not reach a significant difference level. This result indicates that the experimental and control groups were homogeneous in their pre-tests of sustained and selective attention, and therefore, a follow-up comparative analysis can be carried out.

The effect of regular games on the sustained attention and selective attention of female children in the senior class was greater than that of male children. Previous studies have theoretically and logically discussed the promotion of attentional stability (sustained attention), and the finding that the sensitive period for sustained attention is in the pre-school years suggests that the development of attention in the pre-school years is consistent with the physical and mental development of young children. There is theoretical support for rule-based play to promote sustained attention development.

The other dynamic games in this study are also useful for practicing sustained attention, where children need to pay attention to the changing signals of the game and where they want to win and keep their attention on the game. The games chosen were of interest to the children, and when they could not maintain their attention, they would also keep it in the game through the wilful effort if they wanted to win, and after several games of reinforcement, they would transfer it to doing other things, so the post-test children in the experimental group scored significantly higher on sustained attention than the pre-test scores.

## 6. Conclusion

A lightweight network model, BRNet, with binary convolution and traditional convolution operating in parallel was designed to reduce the complexity of the network model parameters. Secondly, a local binary feature extraction method with a dynamic radius sampling strategy is proposed, and an expression area attention mechanism (EAM) is formed for the setting of network channel weights, to achieve an effective fusion of expression area features with depth features. Finally, cross-entropy and L2 loss are designed to quickly achieve accurate classification of expression images. The experimental results show that the recognition accuracy of this method is higher than most CNN methods for facial expression recognition at a single frame image processing speed of 29 ms. The experimental subjects in the experimental intervention phase were mainly young children with low-to-moderate attention scores screened by self-programming. Some papers found that the improvement of attention stability of female children before and after the maze training experiment was significantly higher than that of male children, and the maze game is also a kind of rule game, so this hypothesis is proposed based on this. After excluding children with ADHD tendencies and those who were mentally underdeveloped and had a history of attention training, suitable subjects were selected for the experimental intervention to examine the effectiveness of the intervention scheme. In the convolutional layer of the GCN, a new method of constructing a learnable adjacency matrix is proposed to model an adaptive adjacency matrix by fusing node features and dependency category information, which can effectively alleviate the over- and underpruning problems caused by the rule-based method of pruning the dependency tree, and a bidirectional GCN network is proposed for relationship extraction by combining the directionality of dependency edges. And a combination of AL-GCN and LA-GCG, combining both the attention mechanism and the learnable adjacency matrix, the model exhibits better performance than using the attention mechanism alone and the learnable adjacency matrix alone. [4].

## Data Availability

The data used to support the findings of this study are available from the corresponding author upon request.
